# Undifferentiated Embryonal Sarcoma of the Liver Involving All Major Hepatic Veins Treated by Left Extended Trisectionectomy

**DOI:** 10.1155/2022/9673901

**Published:** 2022-05-30

**Authors:** Reinaldo Fernandes, Klaus Steinbrück, Jan-Peter Périssé, Rodrigo Luz, Renato Cano, Fernanda Cruz-Nunes, Diego Garcia, Rodrigo Diaz, Fernanda Cavalcanti Carneiro, Andrea Velloso, Carlos Frederico Campos, Marcelo Enne

**Affiliations:** ^1^Equipe Multidisciplinar Hepatobiliar – EMHep, Rio de Janeiro, Brazil; ^2^Surgery Department, Antonio Pedro University Hospital, Fluminense Federal University, Niterói, Brazil; ^3^Hepatobiliary Unit, Bonsucesso Federal Hospital, Health Ministry, Rio de Janeiro, Brazil; ^4^Medical School, Antonio Pedro University Hospital, Fluminense Federal University, Niterói, Brazil; ^5^Internal Medicine Department, Antonio Pedro University Hospital, Fluminense Federal University, Niterói, Brazil; ^6^Hepatobiliary Unit, Ipanema Federal Hospital, Health Ministry, Rio de Janeiro, Brazil; ^7^Anesthesiology Department, Clementino Fraga Filho University Hospital, Rio de Janeiro Federal University, Rio de Janeiro, Brazil; ^8^Anesthesiology Department, Pedro Ernesto University Hospital, Rio de Janeiro State University, Rio de Janeiro, Brazil; ^9^Brazilian Hepatology Society, Rio de Janeiro, Brazil; ^10^Pathology Department, Fonte Laboratory, Rio de Janeiro, Brazil

## Abstract

**Introduction:**

Over the past few years, liver surgery has been in constant evolution and gained many improvements that helped surgeons push limits further. A complex procedure such as left extended trisectionectomy, as described by Makuuchi in 1987, may be performed in selected cases.

**Aim:**

Describe a case of successful resection of a huge bilobar liver sarcoma involving all hepatic veins from a young female patient, in which the blood outflow was preserved through an inferior right hepatic vein, leaving only segment 6 as liver remnant. *Case Report.* A 19-year-old female with a 3-month history of abdominal pain, vomiting, and weight loss was referred for our evaluation. CT scan and MRI revealed a heterogeneous and bulky expansive hepatic lesion, sparing only segment 6, with an estimated volume of 530 cm^3^, corresponding to a 1.2 FLR/BW ratio. The tumor involved the three major hepatic veins, but an inferior right hepatic vein was present, draining the spared segment 6. She was submitted to a left trisectionectomy extended to the caudate lobe and segment 7, including resection of all hepatic veins and lymphadenectomy of the hepatic pedicle. She was discharged on the 7th postoperative day without complications. The histopathological and immunohistochemical analysis demonstrated an undifferentiated embryonal sarcoma of the liver.

**Conclusion:**

Inferior right hepatic vein-preserving left extended trisectionectomy is a safe and feasible procedure that should be performed by a hepatobiliary team experienced in major complex hepatectomies.

## 1. Introduction

Over the past few years, liver surgery has been in constant evolution and gained many improvements that helped surgeons push limits further, with good outcomes. Radiological evaluation with magnetic resonance imaging (MRI), volumetric estimation of the future liver remnant (FLR), and liver venous deprivation and a better understanding of the liver anatomy and physiology are examples of these enhancements.

Makuuchi et al. [1], in 1987, pioneered new approaches for resection of tumors involving the right hepatic vein (RHV) due to the presence of an inferior right hepatic vein (IRHV). Since then, many other authors have described the usefulness of the IRHV to perform minor or major hepatectomies when resection of the RHV is necessary [2–5]. In selected cases, extended hepatectomies associated with resection of the three hepatic veins were performed, preserving only segment 6 and the IRHV [2, 4, 6].

Herein, we present a case of successful resection of a substantial bilobar undifferentiated embryonal sarcoma of the liver (UESL), involving all hepatic veins from a young female patient, in which the blood outflow was preserved through an IRHV, leaving only segment 6 as liver remnant.

UESL is an unusual and aggressive primitive mesenchymal cell tumor, responsible for one-tenth of pediatric hepatic malignancies and is the third most common hepatic malignancy in children [7]. To the best of our knowledge, there are only three previous reports in the literature of this type of surgery, and our paper is the first due to UESL (Table 1).

## 2. Case Report

**Figure 1:**
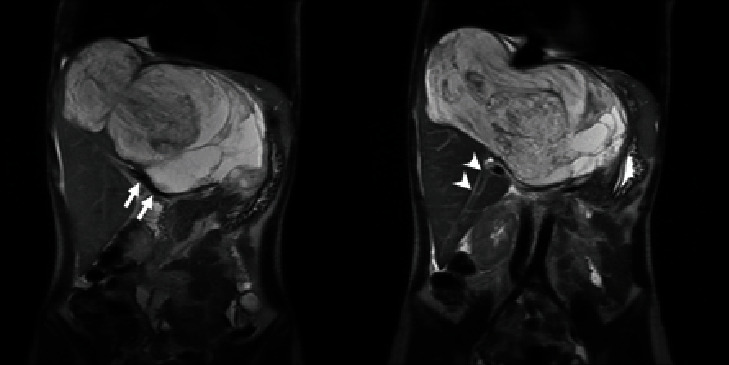
MRI T2-weighted coronal view, showing the huge heterogenous liver mass. The hepatic pedicle (arrows) and segment 6 pedicle (arrowheads) were not involved by the tumor.

**Figure 2:**
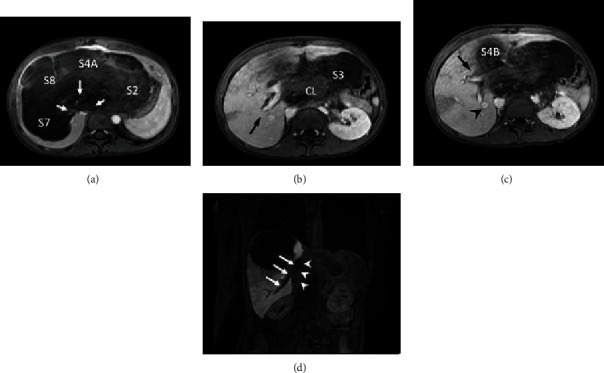
MRI Images. (a) Axial T1 weighted: tumor involvement of major hepatic veins (arrows) and liver segments 2, 4A, 7, and 8. (b) Axial T1 weighted: tumor involvement of segment 3 and caudate lobe; right posterior portal vein is free (arrow). (c) Axial T1 weighted: tumor involvement of segment 4B and the right anterior portal vein (arrow); IRHV entering segment 6 (arrowhead). (d) Coronal T1 weighted, 20-minute hepatobiliary phase: view of the IRHV in segment 6 (arrows) draining into the IVC (arrowheads).

A 19-year-old female was admitted with acute and intense pain in the abdominal upper-left quadrant, associated with nausea and nonbloody vomiting, not responsive to oral medications. Three months earlier, she referred a lighter abdominal pain that spread to the right shoulder and scapula, relieved with oral nonopioid pain medication. By the time, she was weighing 41 kg, having lost 5 kg in the past eight weeks. The patient was using oral contraceptive medication and denied comorbidities, fever, smoking, alcohol intake or other substance abuse, allergies, or previous surgery. She lived in Rio de Janeiro, Brazil, and had no recent travel to endemic areas for infectious diseases. Physical examination revealed a painful and palpable abdominal mass extending from the epigastrium to the left hypochondrium. Laboratory tests demonstrated anemia (hemoglobin 9.4 g/dL [13-18], hematocrit 29.3% [38-52]), slightly elevated liver enzymes, and INR (AST 50 U/L [5-32], ALT 40 U/L [7-31], GGT 321 U/L [8-41], ALP 390 U/L [35-104], and INR 1.5 [<1]), and low plasma albumin (2.9 g/dL [3.5-5.2]). Tumor markers were not altered (AFP 1.3 ng/mL [<10], CA19-9 11.0 U/mL [<37], and CEA 0.5 ng/mL [<3.8]). The abdominal CT scan and MRI showed a lobulated, multilocular, cystic-solid, and heterogeneous expansive hepatic lesion, with a fibrous pseudocapsule, measuring 18 *cm* × 12.1 *cm* × 12.5 *cm*, sparing only hepatic segment 6. Portal or arterial thrombosis was not observed. None of the three hepatic veins could be identified (PRETEXT classification type IVc) [8], but an inferior right hepatic vein was present, with 9.3 mm in diameter, draining the spared segment 6 (Figures 1 and 2). After the hepatobiliary multidisciplinary board discussion, composed of oncologists, radiologists, hepatologists, and hepatobiliary surgeons, we considered that early surgery was the best option, leaving only segment 6 as FLR, once the IRHV could guarantee the blood outflow and considering the following differential diagnoses: ruptured hepatocellular adenoma, atypical hemangioma, and mucinous cystadenocarcinoma. The calculated volume of segment 6 was 530 cm^3^, corresponding to a 1.2 FLR/BW ratio, considered safe for hepatic resection. We did not ponder on neoadjuvant therapy, biopsy, or laparoscopic exploration as the tumor was considered resectable by the team, and no distant metastatic disease was found by thorax and brain CT scan. PET-CT scan was not available before surgery.

We opted to use a transesophageal echocardiogram probe and a Swan-Ganz catheter for cardiac and hemodynamic monitorization during surgery, mainly in case of total vascular exclusion was necessary. We also used a PiCCO catheter (Pulsion System®, Pulsion Medical Systems, Feldkirchen, Germany) in an arterial line to measure the plasma clearance rate of indocyanine green (ICG), to access liver function during hepatectomy.

**Figure 3:**
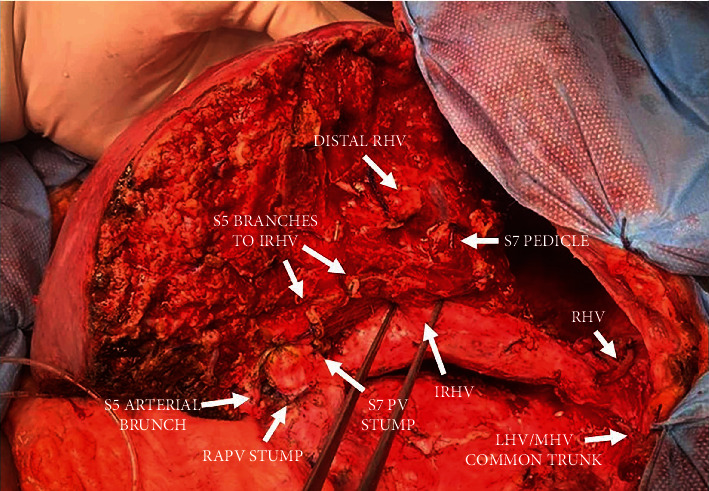
Intraoperative view of segment 6 remnant liver after resection (RHV: right hepatic vein; IRHV: inferior right hepatic vein; LHV: left hepatic vein; MHV: middle hepatic vein; S7 PV: segment 7 portal vein; RAPV: right anterior portal vein; S5: segment 5).

**Figure 4:**
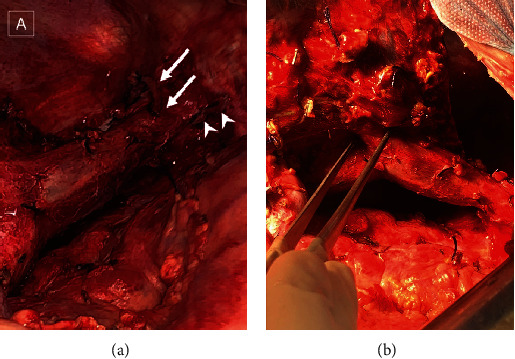
Surgery details—intraoperative view. (a) Inferior vena cava with RHV (arrows) and common trunk of MHV and LHV (arrowheads) divided by vascular stapler. (b) IRHV between forceps.

Surgery was performed with a bilateral subcostal incision together with a midline extension. No peritoneal carcinomatosis or ascites were observed. Initially, we performed a Doppler ultrasonography to confirm the IRHV's patency and the absence of metastasis in the liver remnant. Sequentially, the liver pedicle and the inferior vena cava (IVC) were taped to perform the liver's total vascular exclusion, if necessary. Continuously, we isolated and divided the left portal vein, the left hepatic artery, and the left biliary duct separately. Due to the large volume of tumor load preventing liver mobilization, we opted to perform the liver transection through the anterior approach, using an ultrasonic dissector/aspirator and bipolar diathermy, under Pringle maneuver (five periods of 15 minutes clamping with 5 minutes of clamping-free interval were needed). The right anterior portal pedicle and the portal pedicle to segment 7 were dissected, isolated, and divided, allowing us to identify the demarcation between segments 6 and 5, medially, and between segments 6 and 7, superiorly, on the surface of the right lobe. After liver transection, the tumor mass was detached from the IVC and diaphragm with difficulty, owing to mobilization restriction. Finally, hepatic veins were divided with a vascular stapler (Figures 3 and 4). The patient was submitted to a left trisectionectomy extended to the caudate lobe and segment 7, including resection of all hepatic veins and lymphadenectomy of the hepatic pedicle. The total vascular exclusion was not required. Specimen's surgical margins were free of tumor.

**Figure 5:**
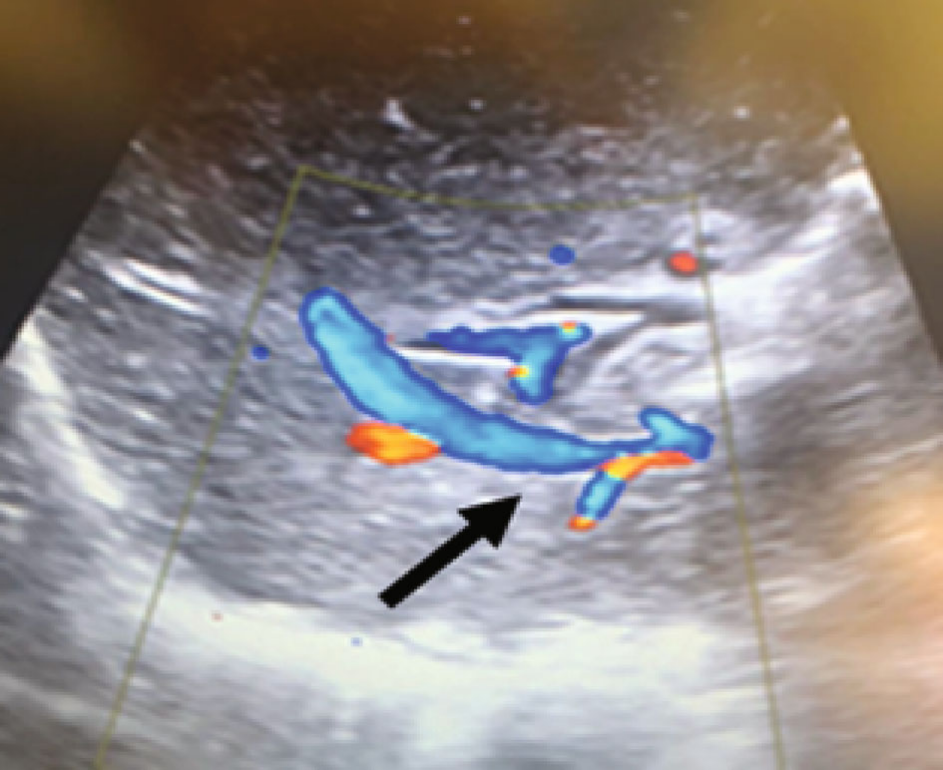
Intraoperative Doppler ultrasonography showing IRHV patency after resection, with hepatofugal flow (arrow).

**Figure 6:**
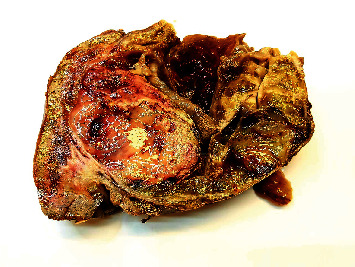
Surgical specimen—analysis confirmed the lobulated, multilocular, cystic-solid, and heterogeneous hepatic tumor, with a fibrous pseudocapsule, measuring 23 *cm* × 12.5 *cm*.

**Figure 7:**
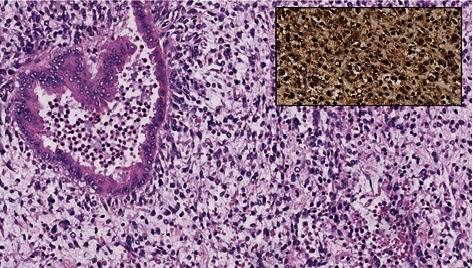
Histopathological exam demonstrating fusiform, oval, or stellate tumor cells distributed over myxoid or fibrous stroma. Nuclear pleomorphism and hyperchromasia are noted. Cell cytoplasm is granular and eosinophilic, with ill-defined cell borders. In the top left, the remnant bile duct is involved by neoplastic cells (H&E, 20x objective). Image insert: strong and diffuse immunostaining for alpha 1-antichymotrypsin (40x objective).

**Figure 8:**
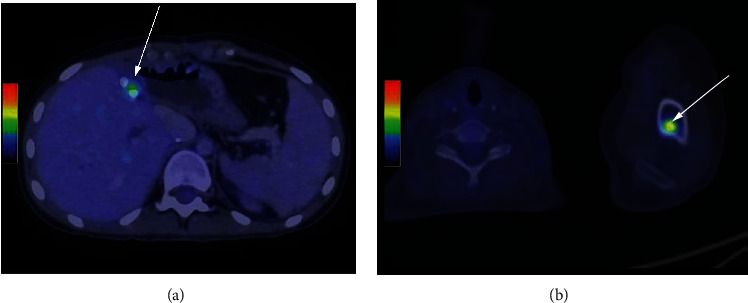
Postoperatory PET scan images. (a) No evidence of disease in the liver (arrow is showing an encapsulated fluid collection). (b) Blastic bone lesion at the left humerus (arrow).

After hepatectomy, blood outflow through the IRHV was rechecked through Doppler ultrasonography (Figure 5). Cholangiography through the cystic duct showed no strictures, and two drains were placed in the abdominal cavity. The mean operative time was 455 minutes, and the mean estimated blood loss was 360 mL, with the administration of one blood unit. She was discharged on the 7th postoperative day without complications. The histopathological and immunohistochemical analysis confirmed positive staining for vimentin, alfa-1-antitrypsin, alpha 1-antichymotrypsin, and Bcl-2 (Figures 6 and 7), which endorsed the diagnosis of UESL. The patient was referred to adjuvant chemotherapy with cyclophosphamide, doxorubicin, vincristine, ifosfamide, and etoposide. She is still in good shape, twenty months after surgery. Although there are no things of disease recurrence in the liver, a recent PET scan identified a blastic lesion at the left humerus, compatible with bone metastasis (Figure 8).

## 3. Discussion

UESL is a rare and very aggressive pediatric malignancy, firstly described in 1978 by Stocker and Ishak [9]. It is responsible for 9-15% of hepatic malignancies in individuals younger than 21 years old, following hepatoblastoma and hepatocellular carcinoma in such range. It mainly affects children from 6 to 10 years old [9]. Also, some studies may show a higher rate in the female population.

Despite showing an 80% mortality rate within 1 year, recent studies have shown a relatively higher long-term survival, most likely due to increased aggressive surgical treatments as described in the present case. The main presenting symptom, when present, is a palpable mass accompanied by abdominal pain. Other symptoms are weakness, anorexia, fever, and vomiting. Imaging exams usually show a large, solid-cystic, and heterogeneous mass with myxoid and necrosis components [10, 11].

Many series [9, 11–14] described, during the past years, different treatment modalities for UESL, such as neoadjuvant and multiagent adjuvant chemotherapy or radiotherapy. Still, they all agreed that radical surgery with clear margins is the best treatment to improve survival. A recent study by Wu et al. [7] shows a significant improvement of overall survival in patients with UESL subjected to aggressive surgical treatment (70.4% 5-year overall survival) if compared to nonsurgical treatment (6.6% 5-year overall survival). In some countries [15]—but not in Brazil—liver transplantation is another option. In addition, the main indication of neoadjuvant therapy is related to unresectable tumors, as it may help reduce tumor bulk and vessel involvement, although there is no standard protocol for such therapy. In our case, after R0 resection of the tumor, the patient received multiagent chemotherapy, as preconized by Memorial Sloan Kettering Cancer Center [11]. She developed febrile neutropenia, which is the most common toxicity with this treatment regimen, but recovered well.

Generally, a liver tumor involving all major hepatic veins is beyond surgical indication. Fortunately, the presence of an IRHV draining the inferior posterior sector of the liver changes this scenario. This accessory vein's incidence varies in the literature, from 21% to 24% [1, 16], and its presence allows isolated segmentectomy [3] and extended trisectionectomy [1, 2, 4, 6]. The adequate venous outflow is one of the keys to avoid hepatic failure or delayed hemorrhage and is essential for the regeneration of the remnant liver, after major resection. From the lessons learned with living donor liver transplantation, we understand that accessory hepatic veins with at least 5 mm in diameter can guarantee satisfactory segmental drainage. In the case described here, the IRHV had almost one cm in diameter, which was considered an adequate caliber for segment 6 outflow. Moreover, the patency of the vein was checked through Doppler ultrasonography preoperatively and twice during surgery (before and after liver resection) to make sure that blood outflow was preserved.

Independent of the primary diagnosis, a type 4 extended trisectionectomy, as described by Makuuchi et al. [1], leaving only segment 6 as FLR, is a rare and complex procedure. To the best of our knowledge, there are only three previous reports in the literature of this type of surgery, and our paper is the first due to UESL (Table 1). Machado et al. [2], in a cholangiocarcinoma case, performed this technique without vein reconstruction and preoperative portal vein embolization (PVE). Kobayashi et al. [4], also in a cholangiocarcinoma case, performed embolization of the right anterior portal branch and portal branch of segment 7 to reach a maximum gain and define the boundary between segments 6 and 7. He also repositioned the confluence of the IRHV in the IVC to prevent outflow blockage. In a recent publication, Yong et al. [6] described this complex procedure in a 9-month-old girl with PRETEXT IVc hepatoblastoma as an alternative for living donor liver transplantation.

In his paper, Makuuchi thought it very difficult to perform such an extended hepatectomy, not only because of the challenging technical aspects of this surgery but also because leaving only one segment of the liver would correspond to a small volume of remnant functional liver parenchyma. Nowadays, we know that leaving only one segment of the liver is not only feasible but also safe [2, 4, 6, 17], if the volume of the liver remnant is adequate. In healthy livers, a minimal of 20% [18] of the standard liver volume (SLV) and a 0.8 FLR/BW ratio are necessary to prevent postoperative liver failure and small-for-size syndrome [19], respectively. Furthermore, Azoulay et al. [20] demonstrated that a FLR of 40% of the SLV was enough to perform safe major hepatectomy for patients who had not only cirrhosis or fibrosis but also liver injury-related chemotherapy. When FLR's volume is not satisfactory, procedures like PVE, as performed by Kobayashi et al., may be necessary to improve the volume of the remnant liver. In the case presented here, no PVE was required, as segment 6 had an estimated volume of 530 cm^3^, corresponding to 57% of SLV and 1.2 FLR/BW ratio (considering a SLV of 932 cm^3^, calculated using the Vauthey et al. formula^21^). The enlargement of this segment could be explained by the obstruction of the major hepatic veins, resulting in increased blood flow through the IRHV, causing augmentation of the vein's caliber, as well as hypertrophy of segment 6. Before surgery, accessing the FLR volume is crucial to perform an extended hepatic resection successfully.

One of the most significant surgical challenges in the case reported here was to initiate the pedicle's dissection due to the tumor's size and to reach out the anatomical limits of segment 6, during our surgical tactical plan. We initiate the parenchyma section in the face of the right portal vein to reach firstly the division of the anterior and posterior branches of the right portal pedicle and then the subdivision of the right posterior portal pedicle to segments 6 and 7. This dissection allowed us to ligate the right anterior portal pedicle and the portal pedicle of segment 7, producing the demarcation lines on the surface of the right lobe to preserve only segment 6 of the liver.

## 4. Conclusion

In conclusion, we can assert that UESL is a rare and aggressive tumor that should be treated aggressively. IRHV-preserving left extended trisectionectomy is a safe and feasible procedure that can be performed in adults or pediatric patients but should be performed by a hepatobiliary team experienced in major and complex hepatectomies. Despite being an aggressive surgical procedure, it may be the only curative option for patients with massive tumors involving the main hepatic veins.

## Figures and Tables

**Table 1:** Data from papers describing type 4 extended trisectionectomy (Dx: diagnostic; IHCC: intrahepatic cholangiocarcinoma; HBlast: hepatoblastoma; Pte: patient; yr: years; m: months; FLR: future liver remnant; SLV: standard liver volume; BW: body weight; NA: not available; PVE: portal vein embolization; Vasc Rec: vascular reconstruction).

Author	Dx	Pte sex	Pte age	FLR/SLV (%)	FLR/BW	PVE	Vasc Rec
Machado, 2008 [2]	IHCC	F	53 yr	38%	NA	No	No
Kobayashi, 2015 [4]	IHCC	M	52 yr	41,7%	NA	Yes	Yes
Yong, 2021 [6]	HBlast	F	9 m	NA	1.8	No	No
Fernandes, 2022	UESL	F	19 yr	57%	1.2	No	No

## Data Availability

No data were used to support this study.

## Abbreviations

MRI: Magnetic resonance imaging
FLR: Future liver remnant
RHV: Right hepatic vein
IRHV: Inferior right hepatic vein
Kg: Kilogram
AFP: Alpha-fetoprotein
CA19-9: Cancer antigen 19-9
CEA: Carcinoembryonic antigen
CT: Computed tomography
FLR/BW: Future liver remnant to body weight
ICG: Indocyanine green
IVC: Inferior vena cava
LHV: Left hepatic vein
MHV: Middle hepatic vein
RAPV: Right anterior portal vein
PV: Portal vein
S: Segment
UESL: Undifferentiated embryonal sarcoma of the liver
PET: Positron emission tomography
Dx: Diagnostic
Pte: Patient
yr: Years
m: Months
IHCC: Intrahepatic cholangiocarcinoma
HBlast: Hepatoblastoma
PVE: Portal vein embolization
SLV: Standard liver volume
Vasc Rec: Vascular reconstruction
RPV: Right portal vein.
